# High c-MET expression is frequent but not associated with early PSA recurrence in prostate cancer

**DOI:** 10.3892/etm.2012.764

**Published:** 2012-10-25

**Authors:** FRANK JACOBSEN, SHARAD NOURAIE ASHTIANI, PIERRE TENNSTEDT, HANS HEINZER, RONALD SIMON, GUIDO SAUTER, HÜSEYIN SIRMA, MARIA CHRISTINA TSOURLAKIS, SARAH MINNER, THORSTEN SCHLOMM, UWE MICHL

**Affiliations:** 1 Institute of Pathology;; 2 Martini-Clinic Prostate Cancer Center and Section for Translational Prostate Cancer Research at the Clinic of Urology, University Medical Center Hamburg-Eppendorf, D-20246 Hamburg, Germany

**Keywords:** c-MET, prostate cancer

## Abstract

c-MET is considered a possible therapeutic target in numerous tumor types and is also a candidate regulator of response to anti-HER2 and anti-epidermal growth factor receptor (EGFR) therapy. The aim of this study was to determine the prevalence and clinical significance of c-MET expression in hormone-naïve prostate cancers. A pre-existing prostate tissue microarray (TMA) containing samples of 4,177 patients treated by radical prostatectomy was used. A total of 3,378 different prostate cancers were successfully analyzed for c-MET expression by immunohistochemistry and follow-up data were available for 4,104 patients. Membranous c-MET immunostaining was performed for 2,655 (78.6%) tumors. High c-MET protein expression was significantly associated with a high Gleason grade (P=0.0018). However, c-MET was not a prognostic marker for biochemical recurrence. c-MET levels were also not associated with other parameters, including tumor stage, nodal stage and surgical margin status. The c-MET protein is often overexpressed in prostate cancer, but has no prognostic relevance. However, the frequent presence of high levels of membranous c-MET protein in prostate cancer cells makes c-MET an attractive target for imaging and treatment.

## Introduction

The receptor protein kinase MET was originally identified as an activated oncogene (1), with hepatocyte growth factor (HGF), also known as scatter factor (SF), as the primary ligand. Usually MET is expressed in a variety of epithelial and mesenchymal cells. MET and HGF have been known to induce cell proliferation, angiogenesis, cell adhesion, invasion, motility and anti-apoptotic responses (2,3). Previous studies have demonstrated a significant MET overexpression in numerous tumor types including glioblastoma (4), melanoma (5), colorectal (6), breast (7), lung (8), gastric (9), thyroid (10) and prostate cancer (11). In addition, aberrant MET expression has been found to be associated with a poor prognosis in a variety of cancers (4,12).

MET expression occurs in the normal prostate epithelium. In contrast to luminal cells, basal cells show consistently high c-MET expression (13). Data on MET expression in prostate cancer are conflicting. Certain studies have reported no associations of high c-MET levels with Gleason grade, while others have reported high MET protein expression in more advanced or metastatic prostate cancer (11,13–15). Recently, c-MET has been proposed as a candidate for targeted cancer therapy in prostate cancer and other tumors (16). In addition, c-MET has been proposed to be a regulator of response to anti-HER2 and anti-epidermal growth factor receptor (EGFR) therapy. For example, in one study, 64 HER2-positive breast cancer patients with increased c-MET expression demonstrated a decreased response to EGFR/HER2 inhibitor therapy (17). Although the data are controversial and mostly negative, the use of anti-EGFR/HER2 therapy remains under discussion for prostate cancer (18).

The aim of this study was to clarify the prevalence and prognostic role of c-MET expression in prostate cancer by using a pre-existing tissue microarray (TMA) including more than 4,000 prostate cancers, the majority with clinical follow-up data. The data show an abundant expression of c-MET and demonstrate that c-MET protein analysis does not serve as a prognostic marker for prostate cancer patients.

## Patients and methods

### Patients

A pre-existing prostate cancer TMA consisting of tissue samples from radical prostatectomy specimens of 4,177 patients, consecutively treated at the Department of Urology and the Martini Clinic at the University Medical Center Hamburg-Eppendorf (Hamburg, Germany) between 1997 and 2008 (Table I), was used in this study. Follow-up data were available for 4,104 patients, ranging from 1 to 150 months (mean, 51 months). None of the patients received neo-adjuvant or adjuvant therapy. Additional (salvage) therapy was only initiated after biochemical relapse (BCR), the clinical end-point of our study. Prostate-specific antigen (PSA) levels were measured quarterly in the first year, followed by biannual measurements in the second and annual measurements after the third year following surgery. Recurrence was defined as a post-operative PSA of 0.2 ng/ml and subsequent increase. The first PSA level above or equal to 0.2 ng/ml was used to define the time of recurrence. Patients without evidence of tumor recurrence were censored at the last follow-up. All prostatectomy specimens were analyzed according to a standard procedure. All prostates were completely paraffin-embedded, including whole-mount sections as previously described (19). A 0.6-mm tissue core was punched out from each sample and transferred to a TMA format as previously described (20). The 4,177 cores were distributed among 9 TMA blocks each containing 129–522 tumor samples. Each TMA block also contained various control tissues including normal prostate tissue and other normal tissues. The utilization of tissues and clinical data was in accordance with the Hamburger Krankenhaus Gesetz (§12 HmbKHG) and approved by our local ethics committee. According to this reputation, informed consent of individual patients was not necessary.

### Immunohistochemistry

Freshly cut TMA sections were stained on 1 day in a single experiment. High-temperature pre-treatment of slides was performed in an autoclave in citrate buffer, pH 7.8 for 5 min. c-MET immunostaining was performed using a monoclonal antibody (clone: EP1454, Abcam, Cambridge, UK, dilution 1:150). The Envision system (Dako, Glostrup, Denmark) was used to visualize the immunostaining. Only membranous staining was evaluated as cytoplasmatic staining, if present, was always linked with stronger membranous staining. The staining intensity (0, 1+, 2+, 3+) and the fraction of positive tumor cells were recorded for each tissue sample. A final score was created from these 2 parameters according to the following scores: negative scores had a staining intensity of 0; weak scores had a staining intensity of 1+ in ≤70% of tumor cells or a staining intensity of 2+ in ≤30% of tumor cells; moderate scores had a staining intensity of 1+ in >70% of tumor cells or a staining intensity of 2+ in >30% but ≤70% of tumor cells or a staining intensity of 3+ in ≤30% of tumor cells; and strong scores had a staining intensity of 2+ in >70% of tumor cells or a staining intensity of 3+ in >30% of tumor cells.

### Statistic analysis

For statistical analysis, the JMP 8.0 software (SAS Institute Inc., Cary, NC, USA) was used. Contingency tables were calculated to determine the association between the c-MET immunostaining score and clinicopathological variables. The Chi-square test was used to identify significant associations. Kaplan-Meier curves were generated for PSA recurrence-free survival. The log-rank test was performed to determine the significance of differences between stratified survival functions. Cox proportional hazards regression analysis was performed to determine the statistical independence and significance between pathological, molecular and clinical variables.

## Results

### Technical issues

A total of 3,378 (81.6%) tumor samples were successfully analyzed in our TMA analysis. The reasons for non-informative cases (762; 19.4%) included a lack of tissue samples or absence of unequivocal cancer tissue in individual TMA samples.

### Immunohistochemistry

**Figure 1:**
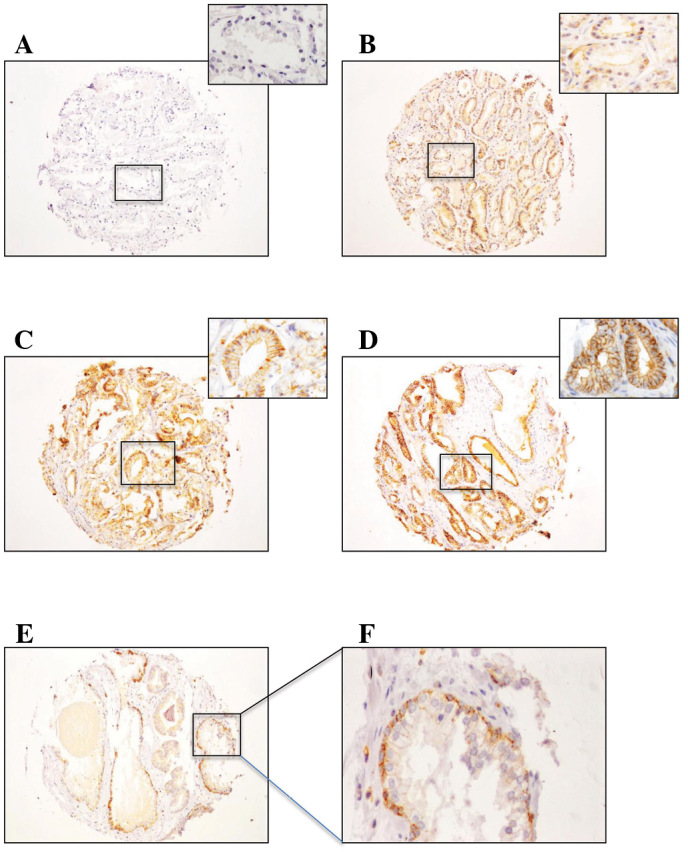
Examples of c-Met immunostaining in prostate cancer and normal tissues. (A) Lack of staining; (B) weak membranous staining; (C) moderate membranous staining; (D) strong membranous staining; (E) strong membranous staining in basal cells; (F) magnification of (E).

**Figure 2:**
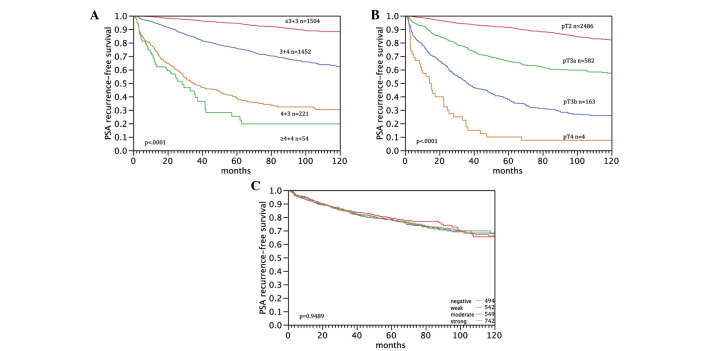
Influence of clinicopathological features and c-MET staining on PSA recurrence. (A) Gleason grade. (B) pT category. (C) c-MET immunostaining. PSA, prostate-specific antigen.

c-MET immunostaining revealed strong membrane staining in our tissues. Although certain cytoplasmic staining was sometimes observed, this was always associated with a markedly higher staining level at the membranes. Membranous c-MET immunostaining was recorded in 2,655 (78.6%) of 3,378 successfully analyzed cases. Staining was weak in 780 (23.1%), moderate in 801 (23.7%) and strong in 1074 (31.8%) prostate cancers (Fig. 1B and C). In benign prostate epithelium, c-MET was always strongly expressed in prostate basal cells (Fig. 1E and F), with a weaker expression in luminal cells as compared with the majority of invasive cancers (Fig. 1A–D). The association between c-MET immunostaining and tumor phenotype is summarized in Table II. Significant associations were demonstrated between strong c-MET expression and high Gleason grade (P=0.0018) as well as increased pre-operative PSA levels (P=0.0064). c-MET staining levels were not associated with tumor stage (P=0.1191), nodal stage (P=0.3907), surgical margin status (P=0.758) or pre-operative PSA level (P=0.0064). Follow-up data were available for 4,104 patients and 3,378 cases were successfully analyzed. As predicted, Gleason grade and pT stage were significantly associated with PSA recurrence in this patient subset (P<0.0001 each; Fig. 2A and B). However, c-MET protein expression levels were not associated with the risk of PSA recurrence (P=0.949, Fig. 2C).

## Discussion

In the present study, the frequency and the potential clinicopathological role of c-MET protein expression was investigated in prostate cancer. The data demonstrate that c-MET expression is abundant in prostate cancer but lacks a clear association with an unfavorable phenotype or a poor clinical outcome. Membranous c-MET staining was observed in 2,655 (78.6%) of 3,378 successfully analyzed cancers and strong c-MET expression was significantly associated with a high Gleason grade (P=0.0018). These results are within the range of previous studies using immunohistochemistry, although the reported c-MET expression levels vary from 33 to 84% (11,13–15). However, the number of analyzed tumors was markedly lower in these studies compared with the present cohort. Pisters *et al*(15) analyzed a cohort of 43 primary prostate cancers. They observed c-MET expression in 84% of cases and revealed an association between c-MET expression and advanced grade prostate cancers (P<0.001). Another group examined 108 prostate cancers and observed c-MET expression in 45% of cases. Their group distinguished between cytoplasmic or luminal membrane staining. No correlation was observed with Gleason grade (11). Watanabe *et al*(14) investigated a cohort of 36 patients, 33% of ‘latent’ and 71% of ‘clinical significant’ prostate cancers displayed cytoplasmic c-MET expression. In total, 38% of low-grade and 80% of high-grade prostate cancers presented c-MET expression. Knudsen *et al*(13) analyzed a cohort of 90 low-grade tumors (Gleason score 6 or 7). c-MET expression was observed in 51% of cancers. They could not identify any correlation between c-MET expression and disease progression. Overall, these studies demonstrate a wide range of c-MET expression rates and are not always consistent with our findings.

It is possible that these controversial data are partly attributable to sampling issues as these studies have all analyzed markedly small patient cohorts and are characterized by highly variable definitions of c-MET-positivity. Watanabe *et al*(14) considered a tumor sample as positive if more than 30% of the tumor cells stained for c-MET, while Humphrey *et al* required only more than 5% to classify a tumor as positive (11). Knudsen *et al*(13) evaluated at least moderate staining intensity as c-MET positive. In addition, the staining of the secretory cells differs from the previous described studies. In the report by Pisters *et al*(15), secretory cell c-MET expression is limited to the central zone. Other studies do not note regional variation of expression (11,14). In contrast Knudsen *et al*(13) did not observe expression of c-MET in secretory cells.

c-MET is frequently expressed in a variety of other cancers. For some cancers, including cholangiocarcinoma, gastric or skin cancer, a clear correlation between c-MET expression level and a poor prognosis has been demonstrated (5,21,22). A skin cancer study revealed significant overexpression of c-MET in all skin cancers with stronger positive responce in malignant melanomas. c-MET expression was stronger in deeper melanomas than in superficial ones (5). However, other investigations identified no significant association between c-MET expression level and clinicopathological parameters (23). Accordingly c-MET could only be used as a prognostic marker in certain cancer types, but not in others.

The high frequency of expression in prostate and other cancer types makes c-MET an attractive potential therapeutic target. Recently, several studies with c-MET inhibitors were realized or are in progress (24,25). Recently published studies demonstrate the anti-proliferative efficacy of c-MET inhibitors in combination with androgen ablation therapy for advanced prostate cancer (16,17). This illustrates that co-targeting of c-MET and androgen signaling pathway might be a therapeutic option for the treatment of prostate cancer in the future (16).

In conclusion, the results of this study reveal that c-MET is frequently overexpressed in prostate cancer. A significant correlation was demonstrated between strong c-MET expression and high Gleason grade, but not with other clinicopathological parameters. Although c-MET appears to be involved in the progression of prostate cancer, this study does not confirm a role of c-MET as a prognostic marker in patients with prostate cancer.

## Figures and Tables

**Table I:** Pathological and clinical data of the arrayed prostate cancers.

	No. of patients^a^	
Variable	Study cohort on TMA (n=4,177)	Biochemical relapse among categories (n=4,104)
Follow-up (months)		
Mean	51.1	-
Median	38.1	-
Age (years)		
<50	119	119
50–60	1,249	1,237
60–70	2,388	2,347
>70	277	260
Pre-treatment PSA (ng/ml)		
<4	631	625
4–10	2,356	2,230
10–20	774	759
>20	225	203
pT category (AJCC 2002)		
pT2	2,789	2,780
pT3a	806	786
pT3b	412	374
pT4	25	33
Gleason grade		
≤3+3	1,593	1,589
3+4	1,847	1,828
4+3	442	426
≥4+4	146	115
pN category		
pN0	1,882	1,840
pN+	146	123
Surgical margin		
Negative	3,255	3,224
Positive	751	717

a Numbers do not always add up to 4,177 in the different categories due to cases with missing data. TMA, tissue microarray; PSA, prostate-specific antigen; AJCC, American Joint Commission of Cancer staging system.

**Table II:** MET expression and tumor phenotype.

Variable	No. of successfully analyzed samples	c-MET immunohistochemistry result				P-value	
		Negative (%)	Weak (%)	Moderate (%)	Strong (%)	
All tumors	3,378^a^	21.4	23.1	23.7	31.8	
Tumor stage						
pT2	2,203	22.2	23.5	22.9	31.4	0.1191
pT3a	695	20.3	20.4	25.0	34.2	
pT3b	335	20.0	26.0	23.9	30.1	
pT4	21	4.8	23.8	19.0	52.4	
Gleason grade						
≤3+3	1,204	24.1	25.0	21.2	29.7	0.0018
3+4	1,567	20.9	21.6	25.1	32.4	
4+3	362	16.6	22.7	26.0	34.8	
≥4+4	118	16.9	26.3	16.9	39.8	
Nodal stage						
N0	1,529	18.3	21.9	24.3	35.5	0.3907
N+	123	23.6	23.6	19.5	33.3	
Surgical margin						
Negative	2,602	21.4	23.5	23.3	31.8	0.758
Positive	632	21.8	21.5	24.4	32.3	
Pre-operative PSA level (ng/ml)						
<4	468	16.9	22.4	25.4	35.3	0.0064
4–10	1,918	20.9	22.7	24.7	31.8	
10–20	645	25.6	24.3	20.6	29.5	
>20	191	26.7	22.5	17.8	33.0	

a Numbers do not always add up to 3,378 in the different categories due to cases with missing data. PSA, prostate-specific antigen.
